# Clinicopathologic characteristics at diagnosis and the survival of patients with medullary breast carcinoma in China: a comparison with infiltrating ductal carcinoma-not otherwise specified

**DOI:** 10.1186/1477-7819-11-91

**Published:** 2013-04-22

**Authors:** A-Yong Cao, Min He, Liang Huang, Zhi-Ming Shao, Gen-Hong Di

**Affiliations:** 1 Breast Cancer Institute, Cancer Centre/Cancer Institute; Department of Oncology, Shanghai Medical College, Fudan University, 270 Dong’an Road, Shanghai, 200032, People’s Republic of China; 2 Department of Oncology, Shanghai Medical College, Institutes of Biomedical Science, Fudan University, 270 Dong’an Road, Shanghai, 200032, People’s Republic of China

**Keywords:** Medullary breast carcinoma, Tumor characteristics, Clinical outcomes

## Abstract

**Background:**

Few studies have addressed the biological features of medullary breast carcinoma (MBC) in the context of clinical outcomes. We sought to compare the baseline demographics, standard pathologic factors and long-term clinical outcomes between MBC and infiltrating ductal carcinoma-not otherwise specified (IDC-NOS) using a large database.

**Methods:**

A total of 2,202 cases with pure IDC-NOS and 188 cases with typical MBC meeting the inclusion criteria were identified. The clinical and biological features, the overall survival (OS) and recurrence/metastasis-free survival (RFS) were compared for both groups.

**Results:**

There were a higher proportion of patients diagnosed prior to 40 years of age in the MBC group compared to the IDC-NOS group. MBC cases demonstrated less aggressive tumor features such as lower tumor stage, smaller tumor size and a lower proportion of nodal involvement than IDC-NOS; however, immunohistochemical staining revealed that MBC displayed the triple-negative phenotype more often than IDC-NOS cases (40.4% versus 26.2%; *P* <0.001). Although the clinical behavior of MBC was not commensurate with its pathologic features, women diagnosed with MBC had a lower frequency of recurrence/metastasis (*P* = 0.032) and death (*P* = 0.042) than those with IDC-NOS, and the 10-year OS and RFS were significantly higher for MBC (91% and 74%) compared to IDC-NOS (81% and 64%). Moreover, multivariate analysis revealed that TNM stage was a statistically significant factor for survival.

**Conclusions:**

MBC in Chinese women demonstrated less aggressive behavior and better prognosis than IDC-NOS. This favorable outcome was maintained after 10 years.

## Background

Medullary breast carcinoma (MBC) accounts for less than 5% of all invasive breast cancers, and usually presents distinct clinicopathologic characteristics from infiltrating ductal carcinoma-not otherwise specified (IDC-NOS) [1]. Histologically, the tumor is characterized by the medullary growth of large cells with a high histological grade (particularly high mitotic count), circumscribed edges, central fibrosis and necrosis, and is often accompanied by lymphocytic infiltration [2].

Medullary carcinoma has long garnered interest due to its morphological and immunophenotypical association with two of the most controversial entities in breast cancer: BRCA1-linked tumors, which often exhibit a much higher rate of MBC and are very frequently estrogen receptor (ER)-negative, and basal-like cancers, which express at least one basal marker [3]. Genetic alterations in the most common tumor suppressor gene, p53, are detected in 50% to 100% of typical MBCs [4]. Moreover, the percentage of human epidermal growth factor receptor 2 (HER2/neu) expression in MBC was reported to range from 10% to 33% [5]. The detection of alterations in these genes would allow us to understand the nature of MBC more clearly and to create new gene therapy strategies in the future.

Despite its aggressive histological features (such as the lack of hormonal receptors and grade of 3), medullary carcinoma has a better prognosis than non-medullary carcinoma. Several studies have reported favorable outcomes for MBC based on relapse-free survival or overall survival (OS) [6-8], and MBC has superior survival compared to IDC-NOS, with 10-year survival rates ranging up to 84% [9]. Furthermore, a comparison of 20-year survival outcome using the data in the published series by Richardson *et al*. found that the 20-year survival rate was 14% better than for non-medullary carcinoma [10].

Although some studies have reported tumors in women with MBC, there is no consensus regarding the clinicopathologic characteristics and long-term outcomes of MBC, or whether it is different from IDC-NOS in terms of prognostic factors. We therefore undertook an extensive comparison of MBC and IDC-NOS using a large database to provide a more complete and reliable assessment of the biological phenotypes and clinical behaviors. The present population-based study elucidated the prognosis of MBC and IDC-NOS in terms of recurrence/metastasis-free survival (RFS) and OS in Chinese women.

## Methods

### Patients and follow-up

We retrospectively reviewed the consecutive data of 188 patients with MBC, ascertained by histopathological examination and treated at the Department of Breast Surgery, Cancer Hospital/Cancer Institute, Fudan University (Shanghai, China), during the period of 1 January 1999 to 1 October 2010. According to the number of patients with MBC who were enrolled every year during the study period, a similar proportion of patients with infiltrating ductal carcinoma (IDC) were randomly selected in the corresponding year, and a total of 2,202 patients with IDC-NOS were recruited and enrolled as controls. Tumors were classified histologically as MBC or IDC-NOS only according to the WHO classification criteria. The patients were all female, without distant metastasis at first diagnosis, and with typical MBC [11] and pure IDC according to the inclusion criteria. MBC was not further subtyped in these databases, and patients with mixed MBC and IDC were excluded. Histological grade and lymphovascular invasion were not analyzed in the present study because in many cases this information was not available.

All patients were required to undertake a complete physical examination, bilateral mammography, chest radioscopy, ECG, ultrasonography of breasts, axillary fossa, cervical parts, abdomen and pelvis, and routine blood and biochemical tests, before surgery and accompanying adjuvant therapy, according to the standards that were used during surgery. All the patients who had relapse risks received adjuvant chemotherapy of different regimens for 4 to 6 cycles followed by local radiotherapy (if required) and/or hormonotherapy (if required), according to the standard therapy at the time of surgery. All the participants gave informed consent. This project was approved by the Scientific and Ethical Committee of the Cancer Hospital of Fudan University.

Follow-up data were collected annually from medical records for breast cancer recurrence, new primary cancers and death. Personal contact with patients, including routine correspondence or telephone visits, was used to follow the patients. The follow-up examinations were carried out at the Cancer Hospital of Fudan University every 3 months during the first 2 years, every 6 months during the next 2 years and once a year thereafter.

### Methods for biological characteristics

The immunohistochemical status of each postoperative paraffin-embedded tumor sample was defined through immunohistochemical staining, including antibodies to ER, progesterone receptor (PR) and HER2/neu (All primary monoclonal antibodies were provided by Dako). All the primary monoclonal antibodies were purchased from Dako (Hamburg, Germany). The detailed staining procedures were performed strictly according to the manufacturer’s instructions. Negative controls were obtained by incubating parallel slides without primary antibodies. Sections known to be stained positively in each run served as positive controls. The percentage and intensity score of stained tumor cells (ER, PR, HER2/neu, p53, cathepsin-D and proliferating cell nuclear antigen (PCNA)) were determined by at least two independent pathologists. The percentage was interpreted as follows: 0, no staining observed; 1, ≤25% of cells with positive staining; 2, 25% to 50%; 3, 50% to 75%; and 4, 75%. In terms of the intensity score, a score of 0 referred to a negative result, 1 to a weakly positive result, 2 to a moderately positive result and 3 to a strongly positive result. The two scores were combined and produced a final score. For all these markers except HER2/neu staining, a score of 0 was defined as negative and 1 to 12 as positive, while strong membranous staining scores of 9 to 12 (DAKO score 3+) were defined as positive.

### Statistical analysis

The association of clinicopathologic factors was evaluated using Pearson’s chi-squared test or Fisher’s exact test. The primary clinical outcomes for this study were RFS and OS. OS was defined as the time from the first diagnosis of primary breast cancer to death from any cause, and RFS was defined as the time from the same starting point to local recurrence or metastasis. Survival time was calculated from the date of surgery to these endpoints, censoring at the date of last contact and non-breast primaries. The 10-year survival rate was calculated using the Life Tables method. Survival curves were obtained using the Kaplan-Meier method, and the log rank test was used to determine the statistical significance in comparative survival for a variety of patient and tumor characteristics. All of the statistically significant variables observed in univariate analysis were investigated by means of multivariate analysis using the Cox proportional hazards model. All *P* values <0.05 were considered statistically significant. All *P* values are two-sided. The SPSS 15.0 software package (SPSS Inc, IBM, Armonk, NY, USA) was used for statistical analysis.

## Results

### Patient characteristics

The MBC group comprised 188 patients between the ages of 26 years and 82 years; the mean age was 52.1 years. The IDC-NOS group comprised 2,202 patients between the ages of 23 years to 90 years; the mean age was 51.8 years. The clinical characteristics of patients in the medullary cohort compared with those of the IDC-NOS control group are presented in Table 1.

**Table 1:** Baseline characteristics and treatment patterns of all patients

**Characteristics**	**MBC**		**IDC-NOS**		***P*value**
	**n = 188**		**n = 2,202**		
	**n**	**%**	**n**	**%**	
Age (years)					
≤40	31	16.5.	290	13.2	0.200
>40	157	83.5	1,912	86.8	
Tumor size					
T ≤2	82	43.4	623	28.3	<0.001
2< T ≤5	102	54.0	1,277	58.0	
T >5	5	2.6	170	7.7	
Unknown	0	0	132	6.0	
Nodal status					
0	132	70.2	1,130	51.3	<0.001
1 to 3	45	23.9	559	25.4	
4 to 10	8	4.3	347	15.8	
>10	3	1.6	110	5.0	
Unknown	0	0	56	2.5	
TNM stage					
I	61	32.4	433	19.7	<0.001
II	117	62.2	1,537	69.8	
IIA	102	54.3	1,253	56.9	
IIB	15	8.0	284	12.9	
III	10	5.3	215	9.8	
Unknown	0	0	17	0.8	
ER status					
Negative	111	59.0	1,167	53.0	0.005
Positive	62	33.0	1,028	46.7	
Unknown	15	8.0	7	0.3	
PR status					
Negative	82	38.0	1,100	50.0	0.005
Positive	119	55.1	1,053	47.8	
Unknown	15	6.9	49	2.2	
HR status					
Negative	94	50.0	785	35.6	<0.001
Positive	79	42.0	1,395	63.4	
Unknown	15	8.0	22	1.0	
HER2/neu status					
Negative	142	68.3	1,512	68.7	0.003
Positive	27	13.0	546	24.8	
Unknown	39	18.8	144	6.5	
Triple-negative					
Yes	76	40.4	550	26.2	<0.001
No	107	56.9	1,610	71.8	
Unknown	5	2.7	42	2.0	
Surgery					
Mastectomy	181	96.3	2,122	96.4	0.949
BCS	7	3.7	80	3.6	
Chemotherapy					
Undo	28	14.9	230	10.4	<0.001
Methotrexate included	73	38.8	857	38.9	
Anthracyclin included	74	39.4	980	44.5	
Taxane included	12	6.4	30	1.4	
Others	1	0.5	10	0.5	
Unknown	0	0	95	4.3	
Radiotherapy					
Undo	168	89.4	1,718	78.0	0.002
Do	18	9.6	402	18.3	
Unknown	2	1.1	82	3.7	
Hormonotherapy					
Undo	125	66.5	1,216	55.2	0.002
Do	60	31.9	961	43.6	
Unknown	3	1.6	25	1.1	
p53					
Negative	52	27.7	214	29.7	0.018
Positive	110	58.5	700	31.8	
Unknown	26	13.8	848	38.5	
Cathepsin-D					
Negative	59	31.4	366	16.6	0.001
Positive	94	50.0	1,022	46.4	
Unknown	35	18.6	814	37.0	
PCNA					
Negative	67	36.4	1,270	57.7	<0.001
Positive	87	47.3	484	22.0	
Unknown	30	16.3	448	20.3	

BCS, breasting-conserving surgery; ER, estrogen receptor; HER2/neu, human epidermal growth factor receptor 2; HR, hormone receptor; IDC-NOS, infiltrating ductal carcinoma-not otherwise specified; MBC, medullary breast carcinoma; PCNA, proliferating cell nuclear antigen; PR, progesterone receptor.

MBCs were much more likely to be smaller on average (43.4% smaller than 2 cm) than IDC-NOS (28.3% smaller than 2 cm; *P* <0.001) at diagnosis. The frequency of node-negative disease was higher for the MBC cases compared to the IDC-NOS cases (70.2% versus 51.3%; *P* <0.001), and the MBC cases were more likely to be diagnosed with TNM stage I disease (32.4% versus 19.7%; *P* <0.001).

The expression of ER was lower in MBC than IDC-NOS: 33.0% and 46.7%, respectively (*P* = 0.005). In contrast, PR had a relatively higher positive rate in MBC cases (*P* = 0.005). Regarding HR (ER or PR) status, the MBC group also had a relatively higher HR-negative rate (*P* <0.001). Of the 169 patients whose HER2/neu status was known, only 27 patients (15.9%) showed HER2/neu over-expression, and there was significant difference in the frequency of triple-negative cases between the MBC group and IDC-NOS group (40.4% for patients with MBC versus 26.2% for patients with IDC-NOS; *P* <0.001). In addition, there were significant differences between the two groups in terms of the amplification status of p53, cathepsin-D and PCNA (p53: *P* = 0.018; cathepsin-D: *P* = 0.001; PCNA: *P* <0.001).

The surgical management of the breast in all patients is illustrated in Table 1. At initial assessment, all patients were offered mastectomy or breast-conserving surgery (BCS) in accordance with clinical guidelines. MBC cases were not different from IDC-NOS cases with regard to the surgical method. A lower proportion of MBC cases received adjuvant endocrine therapy (*P* = 0.002) and no difference in the frequency of adjuvant chemotherapy was observed between the groups (*P* = 0.098). With regard to other treatment methods, less radiotherapy was applied to MBC cases (9.6% in MBC patients versus 18.3% in IDC-NOS patients; *P* = 0.002), probably because of the higher fraction of early-TNM stage patients.

### Univariate survival analysis

The median follow-up was 44.4 months (range, 5 to 139 months), 64.9 months for the MBC group versus 42.9 months for the IDC-NOS group. Contact with 63 patients was lost during the follow-up period.

**Figure 1:**
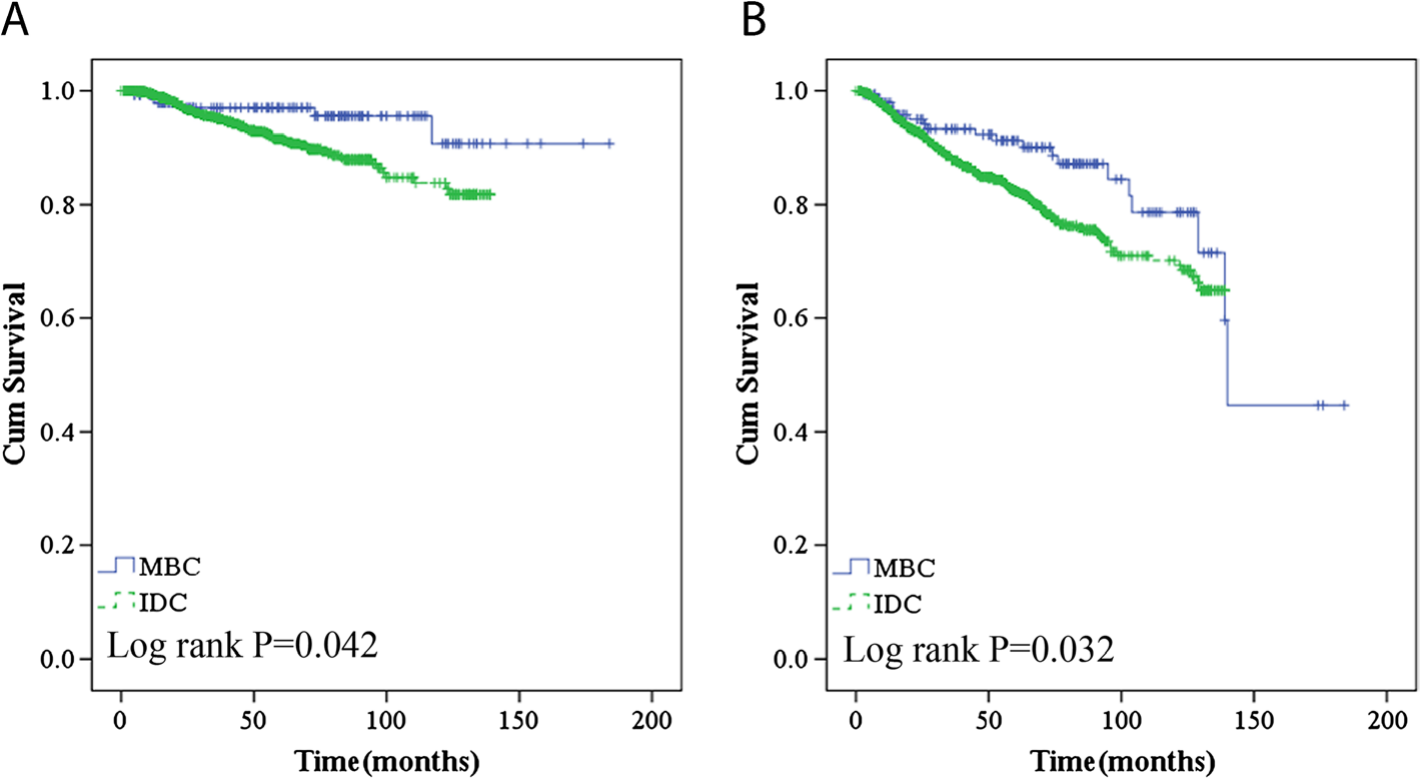
Overall survival (A) and recurrence/metastasis-free survival (B) according to histological type in the general population.

In our cohort, women diagnosed with MBC had a lower frequency of experiencing recurrence/metastasis (*P* = 0.032) and death (*P* = 0.042) compared to those with IDC-NOS. MBC was associated with improved 10-year OS and RFS rates (91% and 74%) compared to IDC-NOS (81% and 64%) (Figure 1A,B) due to its more favorable biological characteristics. However, after adjustment for the common prognostic factors, tumor size and nodal status, we did not observe a survival benefit of the MBC histological type.

We further compared 5-year survival rates (RFS and OS) for T1 triple-negative/node-negative MBC or IDC-NOS cases receiving chemotherapy versus those not receiving chemotherapy, and these same comparisons were also performed in T2 cases (data not shown). The results demonstrated that patients with triple-negative/node-negative MBC or IDC-NOS with a larger tumor size can gain much benefit from chemotherapy.

Univariate analysis for RFS according to clinicopathologic characteristics in MBC revealed that TNM stage was a statistically significant factor for survival (Table 2). The prognostic value of p53 and PCNA amplification status was also evaluated, but neither proved to be a major predictor of worse RFS or OS (RFS: *P* = 0.328 for p53 and *P* = 0.576 for PCNA; OS: *P* = 0.125 for p53 and *P* = 0.425 for PCNA). Furthermore, we found that the adjuvant treatment methods also had an effect on RFS, but these findings had not been further validated in the long-term follow-up and multivariate analysis.

**Table 2:** Prognostic significance of clinicopathologic factors and influence on long-term recurrence/metastasis-free survival in entire cohort and MBC subset

**Covariates**	**Univariate analysis**				**Multivariate analysis cox regression of survival**			
	**MBC**		**Entire cohort**		**MBC**		**Entire cohort**	
	***Χ***^**2**^	***P***	***Χ***^**2**^	***P***	**RR (95% CI)**	***P***	**RR (95% CI)**	***P***
Age ≤50 years	<0.001	0.998	0.47	0.490	1.32 (0.38 to 4.56)	0.666	1.39 (0.91 to 2.12)	0.129
Tumor size ≥2 cm	0.62	0.432	25.35	<0.001	0.42 (0.08 to 2.31)	0.320	2.71 (1.39 to 5.28)	0.003
Node-positive	2.34	0.127	99.36	<0.001	0.74 (0.16 to 3.53)	0.710	2.40 (1.42 to 4.07)	0.001
Higher TNM stage	4.53	0.031	95.60	<0.001	7.84 (0.76 to 80.54)	0.083	2.19 (1.33 to 3.62)	0.002
ER-positive	0.14	0.706	0.57	0.184	1.15 (0.34 to 3.98)	0.817	0.92 (0.58 to 1.44)	0.697
PR-positive	0.04	0.847	0.38	0.103	1.06 (0.28 to 3.92)	0.936	0.80 (0.52 to 1.24)	0.321
HER2/neu-positive	1.70	0.193	8.29	0.004	1.31 (0.37 to 4.69)	0.677	1.31 (0.86 to 2.00)	0.204
Chemotherapy	1.02	0.314	0.89	0.345	0.37 (0.07 to 2.01)	0.251	0.60 (0.30 to 1.20)	0.151
Radiotherapy	7.75	0.005	96.42	<0.001	0.13 (0.03 to 0.71)	0.015	1.81 (1.22 to 2.92)	0.015
Hormonotherapy	0.13	0.722	18.70	<0.001	0.50 (0.13 to 1.93)	0.317	0.69 (0.43 to 1.01)	0.122

CI, confidence interval; ER, estrogen receptor; HER2/neu, human epidermal growth factor receptor 2; MBC, medullary breast carcinoma; PR, progesterone receptor; RR, relative risk.

**Figure 2:**
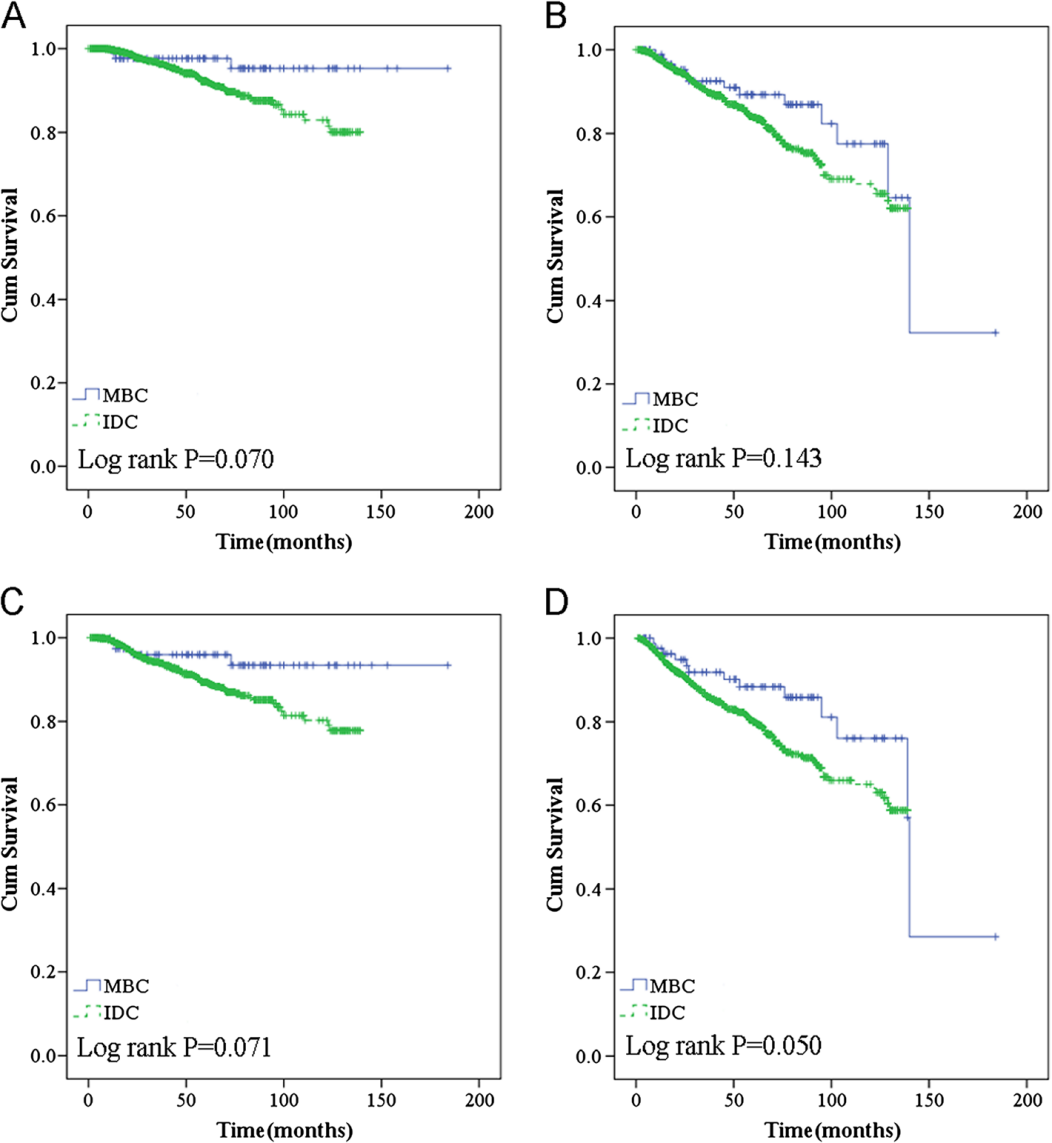
Overall survival in TNM stage II (A) and recurrence/metastasis-free survival in stage II (B) according to histological type in the general population; overall survival (C) and recurrence/metastasis-free survival (D) according to histological type in larger tumor size (≥2 cm) group.

**Figure 3:**
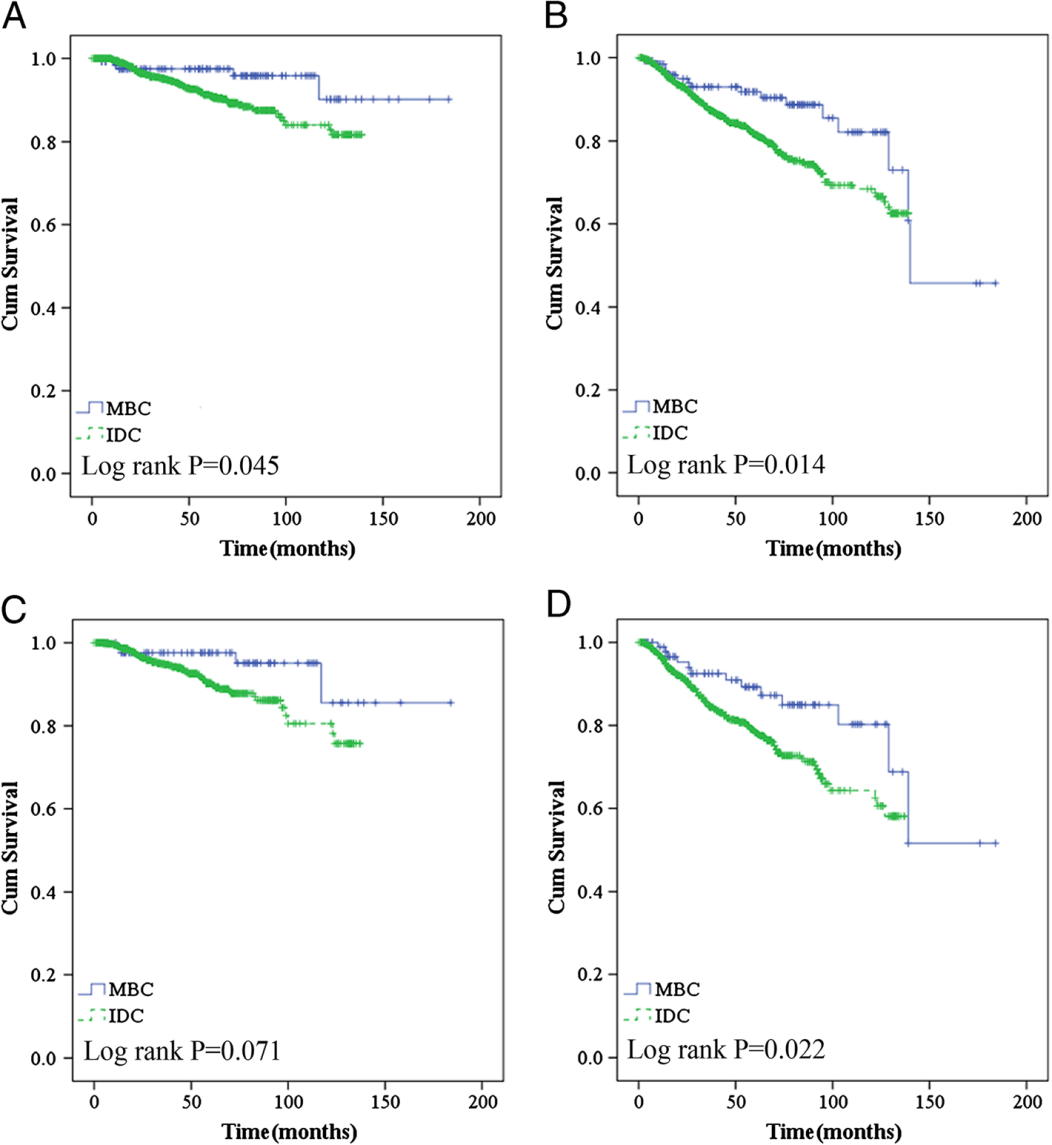
Overall survival (A) and recurrence/metastasis-free survival (B) according to histological type in chemotherapy group; overall survival (C) and recurrence/metastasis-free survival (D) according to histological type in non-hormonotherapy group.

In stage-matched analysis for OS and RFS, MBC showed a trend toward better survival compared to IDC-NOS, especially in the TNM stage II cohort, but this trend was not statistically significant (Figure 2A,B). Moreover, medullary phenotype was also one of the major predictors of better survival in the patients who exhibited larger tumor size (≥2 cm; *P* = 0.050) (Figure 2D). In our cohort, MBC patients who received chemotherapy and did not receive endocrine therapy had a lower rate of recurrence/metastasis or death compared to their counterparts (Figure 3).

### Multivariate survival analysis

Multivariate analysis by Cox regression revealed TNM stage to be the most significant prognostic factor (Table 2). Adjuvant radiotherapy was initially associated with a significant RFS advantage, which was validated in the following multivariate analysis. Specifically, it did not significantly improve the RFS for patients younger than 50 years, with lesions greater than 2 cm in size that were node-positive, ER- or PR-negative, and HER2/neu-positive.

## Discussion

MBC is a type of invasive breast carcinoma that usually has unique characteristics in terms of demographics and clinicopathologic disease. Patients with MBC have continued to garner interest as a distinct disease entity. In this study, we examined the associations and prognostic relevance of medullary histological features in a series of 188 invasive carcinomas compared with IDC-NOS of the breast.

Some interesting findings were observed in our cohort of patients with medullary histology. In addition to a detectable difference in mean age at diagnosis between the study cohort and the control group, a higher fraction of MBCs occurred among the subgroup of young patients, aged 40 years or younger. A higher fraction of MBC cases has previously been linked to poor prognosis [12-14]. Despite its relatively earlier age of onset, these cases of MBC in our cohort showed less aggressive tumor features such as lower tumor stage, smaller tumor size and a lower proportion of nodal involvement than IDC-NOS, which was not consistent with reports that cite a significant association of medullary tumors with adverse prognostic features [15-17]. To our knowledge, few previous studies have described this finding.

Since only a few scattered studies have addressed the biological features of MBC, one of the main objectives of the present study was to more comprehensively characterize its biological phenotype. This report definitively confirms and extends the findings of some previous studies [5-9] which indicated that MBC is significantly more likely to be steroid receptor-negative than IDC-NOS. Our results also demonstrate that MBC is more likely to exhibit a lack of HER2/neu amplification, which shows that medullary carcinomas often display a triple-negative phenotype and might have basal-like biological features. Indeed, we found the expression of basal-associated markers such as basal cytokeratins (CK5/6, CK17 and CK14) and epidermal growth factor receptor (EGFR) in more MBC than IDC-NOS cases (data not shown). Additionally, few reports have evaluated the common tumor suppressor gene p53 or other prognostic markers such as cathepsin-D and PCNA, which reflect tumor cell invasion and growth in MBC, respectively. In the present analysis, positivity for p53 was found almost twice as often in MBC compared with IDC-NOS, and it was similar to the results in some published series reporting that the frequency of p53 mutations ranged from 50% to 100% [4,5]. In comparison to the alterations in cathepsin-D and PCNA, which are not highly correlated to IDC-NOS, almost 50% of tumors classified as MBCs in our study were characterized by the positive expression of these two genes. Based on these findings, MBC in Chinese women seem to be biologically different from IDC-NOS. As a heterogeneous group of tumors, this basal-like class of breast cancer encompasses both poor and good prognostic factors.

Although the clinical behavior of MBC is not commensurate to its pathologic features, it is usually thought to have a more favorable prognosis than IDC-NOS. A population-based analysis from the Surveillance, Epidemiology and End Results (SEER) database of the National Cancer Institute (Bethesda, MD, USA) demonstrated that the 10-year OS rate of MBC was 78%. MBC has superior survival compared to IDC-NOS, with 10-year survival rates of up to 84% in other studies [8]. The prognosis of MBC and the 5-year RFS in our database were also very favorable. This favorable outcome was maintained after 10 years. However, after adjustment for tumor size and nodal status, we did not observe a significant difference between the MBC and IDC-NOS groups, which suggested that the survival advantage of MBC was mainly due to its relatively earlier TNM stage. TNM stage has been validated as the most significant independent prognostic factor in multivariate analysis. Some previous studies have reported that the observed favorable survival was extended up to 15 years during the long-term follow-up period. The relapse-free survival rate was reported to be up to 94.9% in the MBC group, which was significantly better than that of the IDC-NOS group (77.5%; *P* = 0.028) [18]. Our study also clearly showed that 10-year RFS and OS were significantly higher for MBC compared to IDC-NOS, but further analysis of more patients with longer follow-up is warranted to verify the exact favorable effect of MBC in Chinese women. Furthermore, the univariate beneficial prognostic effects of the MBC phenotype appear to be restricted to those cases with a larger tumor size and the majority of patients who received adjuvant chemotherapy or did not receive hormonotherapy. We therefore inferred that MBC might be associated with less aggressive biological behavior than IDC-NOS in some definite subgroups and that MBC achieved better chemosensitivity but lower responsiveness to hormonotherapy than IDC-NOS, owing to the corresponding immunohistochemical profiles.

Previous studies have shown nodal status to be the strongest predictor of disease-specific survival or OS [9]. Steve R *et al*. demonstrated a poorer 10-year survival in MBC patients with nodal involvement (67.5%) compared to those without nodal involvement (81.9%) [8]. Furthermore, in a large study of the effect of nodal involvement on MBC, patients were categorized as those with and those without nodal involvement. Ten-year survival rates for the node-negative patients ranged up to 68.7% to 80.2% [6,19,20]. In the current study, we identified a trend of poorer RFS and OS in MBC patients with nodal involvement, but this did not reach statistical significance and multivariate analysis did not demonstrate an effect (*P* = 0.710). This was probably due to the relatively small sample size and short duration of follow-up. Another limitation was that the pathologic information for lymphovascular invasion and histological grade were excluded from the current analysis; these variables could have had an impaired effect on survival.

\We found that MBCs were treated with BCS as often as IDC-NOS, and we did not identify any recurrence/metastasis or death event at an average follow-up period of 49.6 months. There was recent evidence that the local control rates among patients with medullary and IDC-NOS are comparable [18,21]. Although MBCs are more frequently HR-negative and grade 3, characteristics that are associated with poor prognosis, they are simultaneously of early stage, are HER2/neu-negative and are appropriate candidates for BCS.

## Conclusions

MBC in Chinese women showed less aggressive behavior and better prognosis than IDC-NOS. This relatively large retrospective comparative analysis confirmed that the majority of MBC cases typically present in earlier TNM stages and often display a triple-negative phenotype. TNM stage appeared to be the most significant predictor of worse prognosis. With regards to the choice of surgical procedure, breast-conserving therapy should be preferred over mastectomy in the treatment of early-stage MBC.

## Abbreviations

BCS: breast-conserving surgery; CI: confidence interval; ECG: electrocardiography; EGFR: epidermal growth factor receptor; ER: estrogen receptor; HER2/neu: human epidermal growth factor receptor 2; HR: hormone receptor; IDC: infiltrating ductal carcinoma; IDC-NOS: infiltrating ductal carcinoma-not otherwise specified; MBC: medullary breast carcinoma; OS: overall survival; PCNA: proliferating cell nuclear antigen; PR: progesterone receptor; RFS: recurrence/metastasis-free survival; RR: relative risk; SEER: Surveillance Epidemiology and End Results; TNM: TNM Classification of Malignant Tumours; WHO: World Health Organization.

## Competing interests

There is no any conflict of interest about the study.

## Authors’ contributions

ZMS carried out the study conception and design. AYC was responsible for data collection and manuscript writing. LH, GHD and JW participated in the technique support and results analysis. GYL and JSL participated in the design of the study and performed the statistical analysis. ZZS conceived the study, participated in its design and coordination, and helped to draft the manuscript. All authors read and approved the final manuscript.
